# Treatment of obstructive sleep apnea with oral appliances

**DOI:** 10.1186/2196-1042-14-10

**Published:** 2013-05-23

**Authors:** Andressa Otranto de Britto Teixeira, Luciana Baptista Pereira Abi-Ramia, Marco Antonio de Oliveira Almeida

**Affiliations:** Orthodontic Department, School of Dentistry, State University of Rio de Janeiro, Boulevard 28 de Setembro 157, Rio de Janeiro, 20.551-030 Brazil

**Keywords:** Obstructive sleep apnea syndrome (OSAS), Mandibular advancement oral appliance, Twin block, Placebo, Polysomnography

## Abstract

**Background:**

The purpose of this study was to evaluate the effectiveness of mandibular advancement devices (MADs) for treatment of obstructive sleep apnea syndrome (OSAS) compared with the results obtained with a placebo device in accordance with the following indicators: apnea hypopnea index (AHI) per hour of sleep, apnea index (AI) per hour of sleep, mean oxyhemoglobin saturation, sleep efficiency, and percentage of rapid eye movement (REM) sleep.

**Methods:**

This is a controlled, prospective longitudinal study with a follow-up time of 10.5 months. Nineteen patients (8 females and 11 males) with mean age 48.6 years (SD 9.6) were selected for the study. The sample was randomized in terms of device use, and the evaluation design was double blind. A total of 57 polysomnography tests were studied (at baseline, after the use of a MAD, and after the use of placebo). The following variables were assessed: AHI, AI, mean oxyhemoglobin saturation, percentage of REM sleep, and sleep efficiency. Wilcoxon and Mann–Whitney tests were used for evaluating data (*p* < 0.05).

**Results:**

Reductions from 16.3 to 11.7 in AHI and from 5.7 to 3.8 in AI were observed after MAD use. During the use of placebo, AHI increased from 16.3 to 19.6, and AI from 5.7 to 7.5. The other indexes showed no statistically significant differences.

**Conclusions:**

Treatment with oral appliances, i.e., MADs, can be an effective alternative for mild and medium-to-moderate OSAS, but requires strict monitoring due to differences in individual response to this therapy.

## Background

Obstructive sleep apnea syndrome (OSAS) is characterized by recurrent events of upper airway obstruction during sleep associated with clinical signs and symptoms [1]. Obstruction may involve awakening under the effect of increased respiratory effort and a reduction (hypopnea) or complete cessation (apnea) of airflow in the presence of respiratory movements.

According to the American Association of Sleep Medicine, OSAS diagnosis requires the occurrence of at least five episodes of apnea hypopnea per hour of sleep combined with clinical symptoms, the most important of which are loud snoring and excessive daytime sleepiness [1]. The apnea event is considered when the air flow is interrupted during sleep for a period of 10 s or more, and hypopnea when there is a reduction of at least 50% of the breathing capacity combined with a saturation decrease of the oxyhemoglobin in at least 3%.

This syndrome affects an average 4% of adult males and 2% of adult females in the population, increasing as of the fifth decade of life [2–7]. It is worrisome as it can cause pulmonary hypertension and heart failure [1, 8].

The ideal OSAS treatment, whatever it may be, should be capable of normalizing breathing during sleep, consequently eliminating excessive daytime sleepiness and neuropsychiatric and cardiovascular changes [1]. At the same time, it should provide patients with a good quality of life with no side effects or risks [1, 9].

OSAS treatment modalities range from sleep hygiene, which involves withdrawal from alcohol and other drugs, proper body position, and slimming [1], to surgical procedures such as glossectomy, uvulopalatopharyngoplasty, and maxillomandibular advancement procedures [1, 2, 8]. The most common clinical procedure involves continuous positive airway pressure (CPAP) [1, 3, 10, 11]. Oral appliances have been recommended as a treatment option for being simple to use and non-invasive [12]. These devices are intended to increase the volume of the airways through a mechanical maneuver [13–15].

Several authors stated in their studies [5, 8, 10, 16, 17] that oral appliances are a good alternative for the treatment of snoring and OSAS due to their low cost, relative comfort, and ease of use, which can therefore lead to greater patient compliance. Some issues, however, warrant further substantiation if the therapy is to become an effective and safe alternative for treating these respiratory ailments [18–20]. Among these issues are a correct indication compatible with OSAS severity [15, 21], the diversity of available appliances [12], definition of the basic features these appliances should be able to provide [22], and differences in individual responses to therapy [23–25].

The purpose of this study was to evaluate the improvements obtained with a mandibular advancement device compared with those obtained by a placebo device in accordance with the following indices: apnea hypopnea index (AHI) per hour of sleep, apnea index (AI) per hour of sleep, mean oxyhemoglobin saturation, sleep efficiency, and percentage of REM sleep.

## Methods

### Subjects

Patients were selected by two neurologists certified in sleep medicine. These physicians screened subjects in their private offices based on medical history and evidence of obstructive sleep apnea syndrome by means of overnight polysomnography, in addition to a diagnosis indicating that airflow obstruction was not located in the upper portion of the upper airway (nose or nasopharynx). Based on this diagnosis, whenever they believed a patient could be treated with an oral appliance, he/she was referred for evaluation to the orthodontic clinic of the postgraduate program in Orthodontics at the School of Dentistry, State University of Rio de Janeiro. Before sending to the School of Dentistry, the patients were oriented about the different treatment modalities for OSAS.

Inclusion criteria for this research comprised the need for a diagnosis of mild-to-moderate OSAS, with the exclusion of primary snorers (AHI < 5). Diagnosis was based on overnight polysomnography, considered the gold standard for OSAS diagnosis [26]. The diagnosis of lack of nasal obstruction was done using magnetic resonance imaging.

The following patients were excluded from the study: (a) those who did not have at least eight teeth per arch as they were unable to adequately retain the dental devices, (b) those with severe periodontal problems since the force delivered by the device to the teeth might cause tooth loss, and (c) those with a history of temporomandibular disorders due to the fact that the mechanics deployed by the mandibular advancement device generates tension in the joint that might aggravate this disorder. In this way, patients who were selected had mild to moderate OSAS, without the presence of advanced periodontal disease or TMJ disorders, and with more than eight teeth per arch.

The sample consisted of 19 individuals, 8 females and 11 males. The patients' mean age was 48.6 years (SD = 9.6, min = 32.9, max = 64.6), and the mean body mass index of the sample was 29.7. Eight of these individuals presented with mild OSAS, ten with moderate OSAS, and only 1 patient had a diagnosis of severe OSAS, but was included in the study as he refused to use CPAP and had an AHI value (31.1) very close to the cut-off value between moderate and severe OSAS [1].

### Appliance

**Figure 1:**
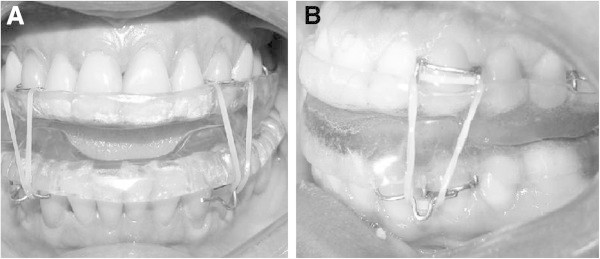
**Twin block appliance in place.** (**A**) Front view and (**B**) side view of the appliance in place.

A twin block (TB) experimental mandibular advancement device was modified for use in this situation. It consisted of two parts, one for the upper arch and one for the lower. It was fabricated from self-curing acrylic resin with occlusal coverage on all teeth so as to reduce changes in tooth positioning that might arise from its use. Each piece had, on its occlusal surface, bilateral slopes with approximately 45° inclination which, when joined, caused the mandible to advance by 75% of each patient's maximum mandibular advancement capacity. These slopes produced an interincisal opening of 8 mm on average. To enhance retention of these devices, four Adams clasps were placed bilaterally on the upper arch (two on the canines and two on the first molars). These clasps could be displaced in the absence of said teeth. Two Adams clasps were placed bilaterally on the first premolars of the lower arch with extensions toward the canines with welded hooks. These clasps, like the ones on the upper arch, could be displaced if these teeth were missing. To ensure that the mandible would remain in an advanced position during sleep, elastics were placed connecting the Adams clasps on the upper canines with the extension hook on the lower clasp. The elastic was medium force, size 3/16 inch (Figure 1A,B).

**Figure 2:**
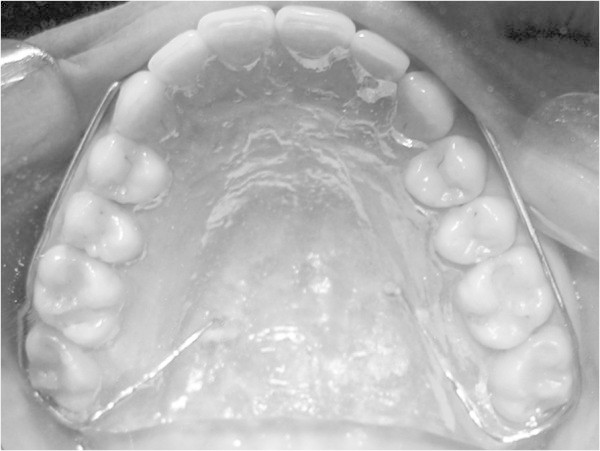
**Occlusal view of placebo device in place.**

The device used as placebo was an acrylic upper plate covering the palate, with a labial arch made of 0.9-mm wire contouring all the teeth and extending past the distal side of the last tooth, where it was fastened to the acrylic plate, in what is known as wraparound device (WRAP) (Figure 2).

### Protocol sequence

This was a prospective longitudinal crossover study with an average 10.5-month follow-up, controlled through the use of a placebo device. The sample was randomized, with the order of use of devices drawn by lot, and the study was double blind since neither the patients nor the doctors who performed polysomnography for assessment of results were aware of the placebo device. The polysomnographies took place in two particular clinics in Rio de Janeiro, Brazil. Both used the same device (Alice model, Philips Respironics, Bothell, Washington, USA).

All patients who participated in the project used both types of devices (experimental and control). Patients were instructed to wear the devices only during sleep, regardless of the time of day. The order of use was randomly chosen by draw. The placebo device was worn for a mean of 3.8 months (SD = 0.8); after which, the patients were subjected to follow-up polysomnography. TB was used for a mean of 6.5 months (SD = 2.0), and overnight polysomnography was performed after this period to assess the results. Before placing the second device, patients spent a week wearing nothing in order to avoid any interference with the results.

This research was submitted to the Ethics Committee of Pedro Ernesto University Hospital and received approval number 1366-CEP/HUPE on December 19, 2005, as it was found to conform to all ethical standards for research in humans, according to resolution No. 196 issued on October 10, 1996 by the National Health Council. All patients signed a free and informed form of consent to participate in the study.

### Tests

The changes produced in OSAS by each appliance were evaluated by comparing the data obtained in the initial polysomnography test and the follow-up polysomnography tests conducted with the use of each appliance at the end of each given period. The following variables were assessed: AHI, AI, mean oxyhemoglobin saturation, percentage of REM sleep, and sleep efficiency.

### Statistical analysis

Paired data were evaluated by the Wilcoxon test. The Mann–Whitney test was used for evaluating unpaired data. A 5% significance level was set for all tests (*p* < 0.05).

## Results

Since this was a crossover study, with patients using both types of appliances in random order, the Mann–Whitney test was performed to check whether the results of each appliance could be analyzed together, regardless of the order in which the appliances were used. The test was conducted at a significance level of 5%.

Comparisons between differences in AHI, AI, mean percentage of O_2_ saturation, percentage of sleep efficiency, and percentage of REM sleep were performed. Table 1 shows the *p* value for each comparison. These analyses led to the conclusion that statistically, the order in which the appliances were placed had no influence on index values, and therefore the two groups could be evaluated together (*n* = 19).

**Table 1:** ***p*** **Value for comparing the order of use of appliances**

Index evaluated	***p*** Value for comparing the order of use of twin block	***p*** Value for comparing the order of use of wraparound (placebo)
AHI	1.0	0.8
AI	0.6	1.0
Mean oxyhemoglobin saturation	0.7	0.7
Sleep efficiency	0.6	0.9
Percentage of REM sleep	1.0	0.4

The first evaluation was carried out using AHI as it is the most widely used index to assess OSAS treatment efficacy. Patient improvement could only be determined by a reduction in AHI greater than or equal to 50%. In addition, to verify normalization, the final AHI should be lower than 5. Tables 2 and 3 (for TB and WRAP, respectively) were constructed based on these criteria.

**Table 2:** **AHI at baseline and after using twin block and percentage reduction of OSAS**

Patient	AHI		Reduction (%)
	Baseline	After use	
1	17.6	8.6	51.3
2	10.2	1.8	82.3
3	15.1	26.1	−76.2
4	20.6	5.3	74.6
5	10.1	19.0	−89.3
6	23.4	17.4	25.6
7	12.7	2.0	84.3
8	13.1	7.9	39.9
9	31.1	4.0	87.1
10	7.9	7.0	11.5
11	23.3	11.6	50.2
12	16.9	16.5	2.3
13	18.9	4.0	78.7
14	22.7	16.4	28.0
15	7.2	17.0	−135.7
16	5.1	6.1	−19.6
17	21.0	3.5	83.6
18	25.0	9.0	64.0
19	7.7	37.9	−394.5
Mean	16.3	11.7	2.5
SD	7.2	9.4	115.6

**Table 3:** **AHI at baseline and after using wraparound (placebo) and percentage reduction of OSAS**

Patient	AHI		Reduction (%)
	Baseline	After use	
1	17.6	20.4	−15.4
2	10.2	4.0	60.6
3	15.1	7.5	50.3
4	20.6	32.4	−56.9
5	10.1	18.0	−79.3
6	23.4	42.3	−81.3
7	12.7	32.5	−155.7
8	13.1	2.3	82.8
9	31.1	19.4	37.6
10	7.9	13.1	−65.3
11	23.3	0.9	96.0
12	16.9	25.6	−51.7
13	18.9	11.3	40.2
14	22.7	39.0	−71.8
15	7.2	1.4	81.0
16	5.1	9.6	−88.2
17	21.0	14.2	33.8
18	25.0	51.0	−104.0
19	7.7	27.8	−262.5
Mean	16.3	19.6	−28.9
SD	7.2	14.8	93.5

**Figure 3:**
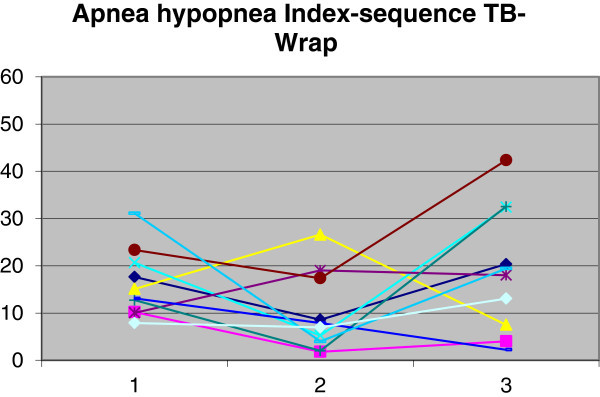
**Demonstration of AHI changes in group 1.** Time 1, baseline; time 2, with twin block; and time 3, with wraparound.

**Figure 4:**
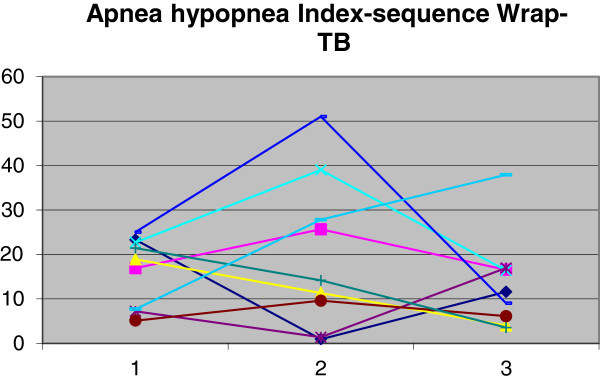
**Demonstration of AHI changes in group 2.** Time 1, baseline; time 2, with wraparound; time 3, with twin block.

The use of TB produced a reduction in AHI from 16.3 (SD = 7.2) to 11.7 (SD = 9.4). The Wilcoxon paired test at 5% was used for data analysis. There was no significant difference (*p* > 0.05) between the group's initial and final means. The use of WRAP yielded an increase in AHI from 16.3 (SD = 7.2) to 19.6 (SD = 14.8). There was also no statistically significant difference (*p* > 0.05) between the means at the two times (T1 and T2). A comparison was made between the reductions in AHI produced by TB and WRAP using the Mann–Whitney test at 5%, which revealed no statistically significant difference between the two (*p* = 0.2). Variations in AHI for each patient are shown in Figures 3 and 4.

Changes in AI were evaluated in the same manner as changes in AHI. Considering these criteria, Tables 4 and 5 were constructed for TB and WRAP, respectively.

**Table 4:** **AI at baseline and after using twin block and percentage reduction of OSAS**

Patient	AI		Reduction (%)
	Baseline	After use	
1	6.4	0.3	95.2
2	2.3	0.9	60.9
3	2.1	8.9	−322.4
4	4.9	0.8	84.7
5	1.6	10.4	−560.5
6	16.3	8.6	47.5
7	6.3	0.5	92.0
8	2.1	1.1	50.5
9	9.3	0.3	96.8
10	1.2	1.3	−11.8
11	3.1	2.7	12.3
12	4.1	12.1	−195.9
13	13.9	0.6	95.8
14	8.1	8.7	−6.8
15	2.0	11.3	−454.7
16	1.9	2.9	−53.9
17	3.7	0.1	96.5
18	15.2	0.7	95.6
19	3.3	0.6	82.3
Mean	5.7	3.8	−36.6
SD	4.8	4.4	199.7

**Table 5:** **AI at baseline and after using wraparound (placebo) and percentage reduction of OSAS**

Patient	AI		Reduction (%)
	Baseline	After use	
1	6.4	6.0	6.4
2	2.3	0.4	84.1
3	2.1	0.4	82.9
4	4.9	11.6	−137.1
5	1.6	6.3	−301.9
6	16.3	22.6	−38.4
7	6.3	23.2	−269.9
8	2.1	0.5	75.7
9	9.3	0.8	91.3
10	1.2	2.6	−119.3
11	3.1	0	100.0
12	4.1	11.2	−172.4
13	13.9	0.6	95.9
14	8.1	13.7	−68.5
15	2.0	0.7	66.0
16	1.9	2.4	−25.7
17	3.7	0.6	84.3
18	15.2	26.0	−71.0
19	3.3	13.0	−296.0
Mean	5.7	7.5	−42.8
SD	4.8	8.7	139.5

**Figure 5:**
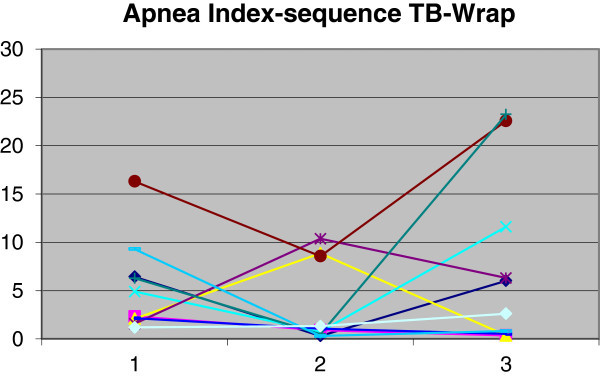
**Demonstration of AI changes in group 1.** Time 1, baseline; time 2, with twin block; time 3, with wraparound.

**Figure 6:**
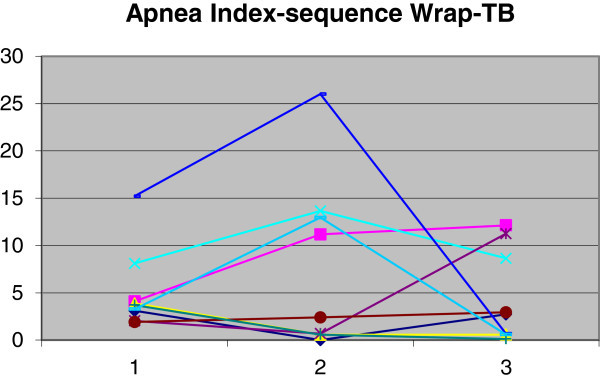
**Demonstration of AI changes in group 2.** Time 1, baseline; time 2, with wraparound; time 3, with twin block.

The use of TB produced a reduction in AI from 5.7 (SD = 4.8) to 3.8 (SD = 4.4). Use of WRAP yielded an increase in AI from 5.7 (SD = 4.8) to 7.5 (SD = 8.7). The Wilcoxon paired test at 5% was employed for data analysis. There was no significant difference (*p* > 0.05) between the means at the two times (T1 and T2) for either device. A comparison was made between the index reductions produced by each device using the Mann–Whitney test at 5%, which revealed no statistically significant difference between AI reductions (*p* = 0.4). Variations in AI for each patient are shown in Figures 5 and 6.

**Figure 7:**
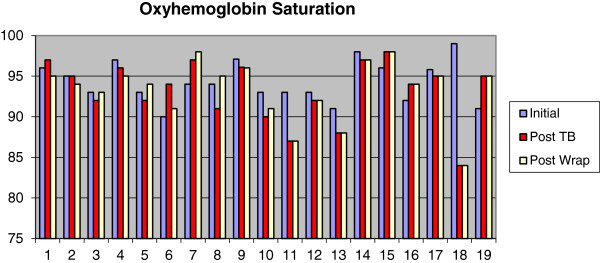
**Measurements of mean oxyhemoglobin saturation at baseline, after twin block and after wraparound.**

**Figure 8:**
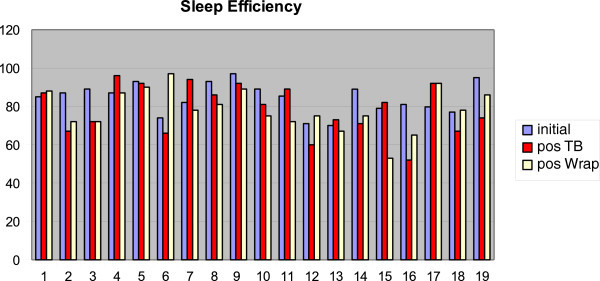
**Measurements of sleep efficiency at baseline, after twin block and after wraparound.**

**Figure 9:**
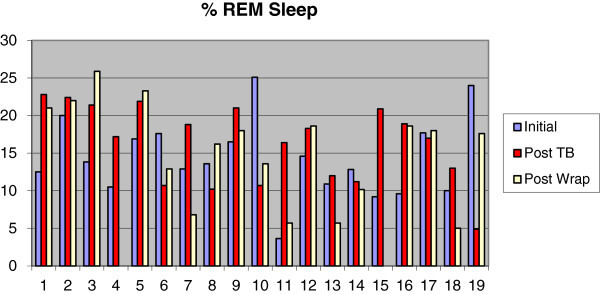
**Measurements of REM sleep at baseline, after twin block and after wraparound.**

The use of TB caused mean oxyhemoglobin saturation to decrease from 94.3% (SD 2.5%) to 93.2% (SD 3.8%), sleep efficiency to drop from 84.4% (SD 7.9%) to 78.6% (SD 12.8%), and percentage of REM sleep to rise from 14.0% (SD 5.0%) to 16.0% (SD 5.0%). The use of WRAP caused oxyhemoglobin saturation mean values to remain unchanged, with differences found only in standard deviation. Sleep efficiency was reduced from 84.4% (SD 7.9%) to 78.5% (SD 10.9%), and the percentage of REM sleep mean values also remained unchanged, with differences found only in standard deviation (Figures 7, 8, and 9).

The Wilcoxon paired test at 5% was employed for data analysis of the means at T1 vs. T2. No significant difference (*p* > 0.05) was found between the means of the two times for either device. A comparison between the results achieved with the two devices was performed by means of the Mann–Whitney test at 5%, which also showed no statistically significant difference.

## Discussion

The results of this study demonstrated clinically that the twin block mandibular advancement device (TB) can be an alternative treatment for OSAS, corroborating other studies which found that oral appliances have indeed emerged as an alternative treatment for this syndrome [1, 5, 12–15, 25, 27]. Mandibular advancement devices are more often indicated than CPAP given the former's greater comfort, which theoretically would tend to increase patient compliance and satisfaction leading to greater adherence to treatment [20], in addition to lower treatment costs. All these factors hinder the use of CPAP, despite its proven greater effectiveness.

The use of a modified TB was based on issues such as cost, ease of fabrication, and greater mandibular motion. The cost, as reported by Eckhart [22] and Lawton et al. [28], should be taken into account because nowadays, OSAS is considered a public health problem [29, 30]; before, it is prescribed and applied to a large portion of the population, it should become affordable. For this reason, it is important that TB be easy to manufacture so it can be easily produced by a large number of laboratories, requiring no training or very specific materials, which could lead to increased costs and access difficulties. Mandibular freedom of motion is yet another factor that makes TB rather appropriate for use in these situations. Mandibular freedom [12, 31, 32] fosters patient comfort, both because patients do not feel that their mouth is stuck and because it offers a certain degree of joint comfort, since protrusion force in itself tends to cause discomfort. Use time evaluated in the study was approximately 6.5 months since the goal was to evaluate long-term results and not just immediate results. This type of evaluation was considered more appropriate because short-term evaluation [4, 18, 19, 28, 33–38] might produce short-lived results, whereas this therapy should last a whole life time. Two main factors are likely to yield different assessments, depending on how long the appliance is used: Airway enlargement caused by muscle stretching and a reduction in upper airway edema [14, 15]. WRAP was chosen as placebo as it does not cause any changes in mandibular or tongue posture that might interfere with the dynamic upper airway architecture and might, therefore, fail to act as a true placebo. Such was the case of the appliance used for this purpose by Blanco et al. [33], Cooke and Battagel [34], Hans et al. [35], and Johnston et al. [36] which, be it due to its size or even because it induced changes in mandibular position by virtue of the opening it produced, can have a real impact on the natural positioning of the structures. This might raise doubts as to whether or not the changes promoted by the appliance did account for the observed alterations. The time frame for placebo evaluation was set for after TB use. Assuming that TB could have reduced the airway edema, a minimum time period would be required to allow the original conditions to be restored. Average TB use time for assessment was approximately 4 months.

This study was undoubtedly limited by a small sample size, which proved unable to demonstrate results statistically. All previous investigations that conducted this type of assessment had equally limited samples [16, 20, 28, 34, 36, 37]. Any research conducted with small samples, although predominant in this type of study, will only reveal clinical trends without showing statistically significant differences. This is mainly due to variations in individual responses, which in small samples tend to confuse investigators. A systematic review of the Cochrane Library [39] found only six studies that used placebos with appropriate methodology, all with small sample sizes ranging from 15 to 28 subjects [19, 33–36, 40] and likewise were only able to show clinical trends. The findings of this study are therefore relevant despite the lack of statistical significance. In seeking to contribute to research aimed at validating this sort of therapy, this was a prospective, longitudinal, randomized and double-blind crossover study with a placebo group. A 5% significance level with 100% probability was set for statistical analysis. To assess patient improvement, the most stringent evaluation criteria ever employed in previous studies was adopted. Patient condition was only considered better if it showed a reduction of at least 50% of the initial index and considered normal if the index was lower than five, which was also considered in the studies by Almeida et al. [16], Blanco et al. [33], Chan et al. [14], Clark et al. [3], and Mehta et al. [19].

Based on this assessment, 47% of patients using TB showed improvement in their condition and 26% had a normal AHI, the most widely used index for treatment evaluation. The index mean fell from 16.3 to 11.7. Moreover, 26% of patients using WRAP improved, and 21% had their OSAS condition normalized, with AHI < 5. The index mean, however, rose from 16.3 to 19.6.

The improvement rate achieved through TB use is within the limits reported in the literature [41] and could have been even better if less stringent standards had been applied. For several researchers [7, 17, 20, 25], an AHI below 10 should be considered as good response, although for Liu et al., [41], a reduction of 25% characterizes a partial response. According to Lee et al. [15], Schmidt-Nowara et al. [42], and O'Sullivan et al. [21], any treatment capable of reducing this index to a value below 20 can be deemed satisfactory. If an index reduction, be it of any magnitude, can be considered an improvement, this sample would have an index of 73.7%.

Although neither index has shown statistical significance, a joint analysis of the indices suggests that TB may be a clinical alternative for OSAS treatment. Nevertheless, further investigation is clearly warranted. Other similarly well-controlled studies should be conducted but with larger samples. Studies with small samples are prevalent in this area due to difficulties in obtaining and controlling the sample [20, 28, 34, 36, 39]. Only larger samples would detect specific differences in evaluation methods, and in the non-homogeneity of the sample, factors which are extremely difficult to control in an OSAS sample.

Above and beyond the authors' belief that this can be an effective treatment, this study shows that variations in individual response are substantial [12, 19, 20, 28] and that it is not possible to establish any specific treatment without a strict control of individual responses, as has been emphasized by the American Association of Sleep Medicine [43]. This control can only be accomplished by polysomnography, despite shortcomings inherent in this test [13]. Individual improvement reports are not acceptable since subjective improvements not always match objective improvements [19, 35], and systemic changes produced by OSA may continue to progress if the condition is not controlled, even if the patient is feeling better.

## Conclusions

The AHI and AI showed a decline in the mean value with mandibular advanced device use and showed increase with placebo use. For the mean oxygen saturation, the mandibular advancement device produced a drop at the index while the placebo kept. Both devices produced worsening in sleep efficiency, and the mandibular advanced device was able to improve the percentage of REM sleep while placebo not. All indexes did not show statistically significant difference and showed quite a difference between patients. It can be concluded that treatment with mandibular advancement oral appliances can be an effective alternative but requires strict monitoring due to differences in individual responses to this therapy.
